# To: Sedative defined daily dose: suggestion for a new monitoring tool

**DOI:** 10.62675/2965-2774.20260037

**Published:** 2026-05-18

**Authors:** Ferhan Demirer Aydemir

**Affiliations:** 1 Çanakkale Onsekiz Mart University Hospital Division of Intensive Care Medicine Department of Internal Medicine Çanakkale Turkey Department of Internal Medicine, Division of Intensive Care Medicine, Çanakkale Onsekiz Mart University Hospital - Çanakkale, Turkey.

TO THE EDITOR,

We read with great interest the recent Research Letter by Noritomi et al. proposing the use of sedative defined daily dose (DDD) as a monitoring tool in critically ill patients.^(1)^ The pursuit of standardized metrics to quantify sedative exposure is both timely and relevant, particularly in an era increasingly focused on sedation stewardship and outcome-oriented intensive care practice. At the same time, several clinical and methodological considerations warrant attention when interpreting sedative DDD beyond its descriptive role.

The DDD concept was originally developed for population-level drug utilization analyses rather than for the assessment of individual patients.^(2)^ In the intensive care unit, sedative dosing is inherently dynamic and driven by rapidly evolving clinical conditions, including illness severity, ventilatory requirements, agitation, *delirium*, and procedural needs. Consequently, similar cumulative DDD values may reflect markedly different clinical trajectories, limiting the interpretability of DDD when used in isolation.

Another important issue relates to sedation depth and its temporal evolution. Cumulative exposure metrics do not capture transitions between deep and light sedation, despite robust evidence linking sedation depth and duration to outcomes such as *delirium* and mechanical ventilation duration.^(2–5)^ Without incorporating sedation targets or time-weighted exposure, DDD-based comparisons may oversimplify complex sedation practices that vary across patients and units.

Furthermore, sedative agents differ substantially in pharmacodynamic properties, clinical indications, and adverse effect profiles.^(4,5)^ Aggregating heterogeneous drugs into a single DDD-based metric may obscure clinically meaningful distinctions, particularly when comparing units with different sedation strategies or protocol preferences. This consideration is especially relevant in patients requiring prolonged mechanical ventilation or those with neurological vulnerability.^(5)^

Finally, the timing of sedative exposure deserves particular emphasis. Early high-dose sedation during acute physiological instability may have different clinical implications than sustained moderate sedation during recovery.^(3)^ A single cumulative metric may therefore mask temporal patterns that are central to clinical interpretation and may explain associations with patient-centered outcomes.

Taken together, sedative DDD appears well-suited as a descriptive indicator of sedative utilization at the unit or health system level. Caution is warranted, however, when extending its use to infer sedation intensity or quality at the bedside. Integrating DDD with measures of sedation depth, temporal patterns, and clinically relevant outcomes may enhance its interpretability and strengthen its role in critical care research.

## References

[1] Noritomi DT, Melo WP, Tavares MS (2026). Sedative defined daily dose: suggestion for a new monitoring tool. Crit Care Sci.

[2] Kress JP, Hall JB (2006). Sedation in the mechanically ventilated patient. Crit Care Med.

[3] Shehabi Y, Bellomo R, Reade MC, Bailey M, Bass F, Howe B (2012). Sedation Practice in Intensive Care Evaluation (SPICE) Study Investigators; ANZICS Clinical Trials Group. Early intensive care sedation predicts long-term mortality in ventilated critically ill patients. Am J Respir Crit Care Med.

[4] Barr J, Fraser GL, Puntillo K, Ely EW, Gélinas C, Dasta JF (2013). American College of Critical Care Medicine. Clinical practice guidelines for the management of pain, agitation, and delirium in adult patients in the intensive care unit. Crit Care Med.

[5] Pandharipande PP, Ely EW (2006). Sedative and analgesic medications: risk factors for delirium and sleep. Crit Care Clin.

